# Linking Inferred Laboratory‐Derived Temperature Stress to the Immunocompetence of Wild *Octopus maya* (Mayan Octopus) G.L. Voss & Solís, 1966

**DOI:** 10.1002/ece3.70805

**Published:** 2025-03-19

**Authors:** Luis Enrique Angeles‐Gonzalez, Laura Alvarez‐Lee, Luis Osorio‐Olvera, Estefany López‐Ripoll, Fernando Díaz, Carlos Rosas, Honorio Cruz‐López, Cristina Pascual

**Affiliations:** ^1^ Laboratorio de Ecofisiología de Organismos Acuáticos Departamento de Biotecnología Marina Centro de Investigación Científica y de Educación Superior de Ensenada, (CICESE) Ensenada Baja California Mexico; ^2^ Departamento de Ecología de la Biodiversidad, Instituto de Ecología Universidad Nacional Autónoma de México Ciudad de México Mexico; ^3^ Unidad Multidisciplinaria de Docencia e Investigación, Facultad de Ciencias Universidad Nacional Autónoma de México Sisal Yucatán Mexico

**Keywords:** aerobic scope, eco‐immunology, immune system, oxygen capacity–dependent thermal tolerance, stressful temperatures, thermal metabolic scope, thermal niche

## Abstract

The “oxygen capacity–dependent thermal tolerance” (OCLTT) hypothesis suggests that the ability of ectotherms to tolerate heat is limited by their ability to supply oxygen to their tissues at various temperatures set by the capacity of the cardiovascular and respiratory systems. Optimal temperatures and oxygen can supply enough energy through adenosine triphosphate (ATP) via the electron transport chain to support fitness‐related processes. Conversely, stressful temperatures indicate an energetic limitation that could describe physiological parameters and biogeographical patterns. Our study aimed to determine if stressful temperatures could be related to immunological performance under a macroecological approach. To prove this hypothesis, we recapitulated key immune parameters, including total hemocyte count, hemagglutination, phenoloxidase system, and lysozyme activity, of wild mayan octopus ( *Octopus maya* ), an endemic species in Mexico's Yucatan Peninsula, with physiological data via thermal metabolic scope (a proxy of the aerobic scope) from its fishing regions. Our results indicate that stressful temperatures (> 27°C) are associated with depression in the immunocompetence of the mayan octopus. Specifically, we found that favorable temperatures (< 27°C) are positively correlated with a better immunocompetence of wild octopus. This study provides evidence that temperature stress inferred from laboratory studies presents a potential tool to determine wild populations' health. However, predictions and modeling should consider additional factors such as demographic distribution, seasonality, biotic/abiotic interactions, and ontogenetic development.

## Graphical abstract

We established a correlation between thermal stress (an aerobic scope proxy) and the immune system in a wild octopus population. This suggests that oxygen capacity–related thermal tolerance may influence immunocompetence in marine species. We highlight potential connections between aerobic scope proxies and immune function.

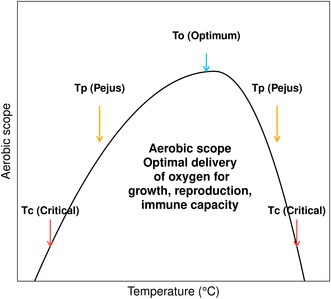

## 1 Introduction

Laboratory studies have utilized respiratory metabolism to investigate how environmental conditions influence organisms' production, storage, and ATP (adenosine triphosphate) consumption processes. These metabolic reactions are crucial for maintaining optimal fitness. The temperature has been particularly identified as a critical factor in the form of a molecular accelerator in biochemical reactions and its impact on increasing energy production (Angilletta 2009). The “oxygen capacity–dependent thermal tolerance” (OCLTT) relationship has gained popularity as an explanation for the relationship between metabolism and temperature. This concept is based on the interdependence between temperature and oxygen requirements at the mitochondrial level to satisfy energy demands (Pörtner 2002, 2012; Pörtner and Peck 2010).

**FIGURE 1:**
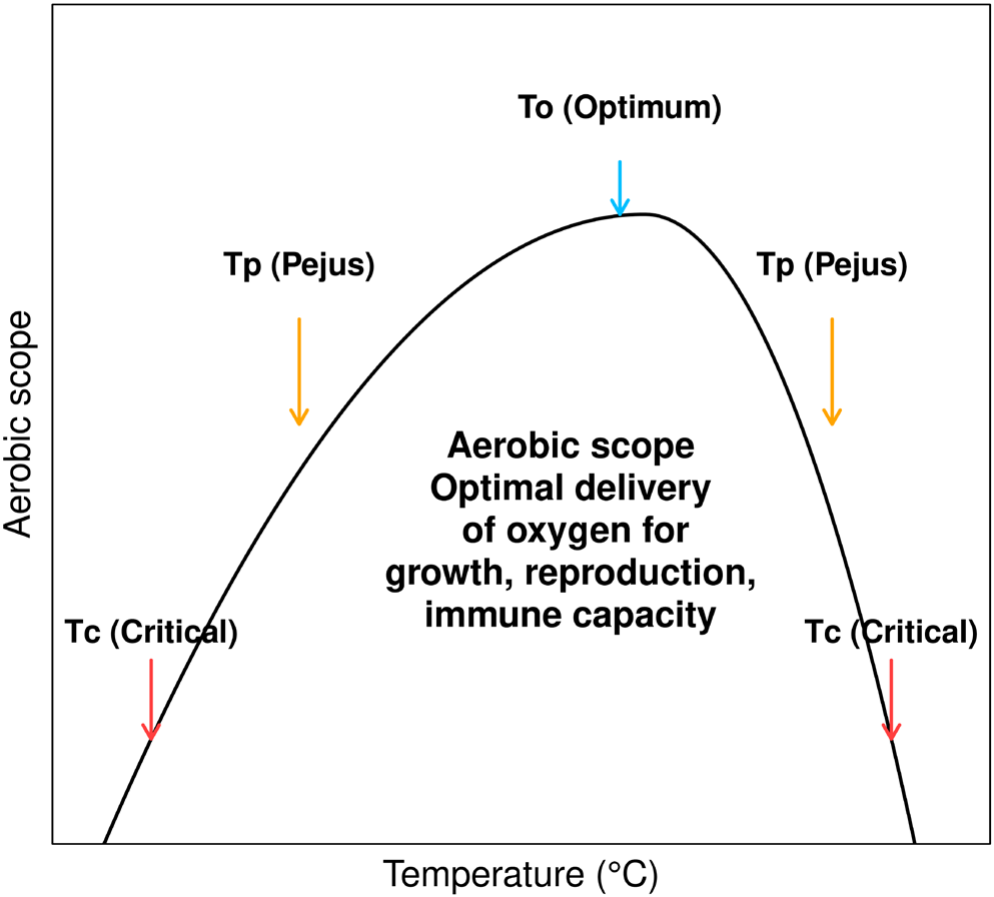
The aerobic scope (AS) peaks at the optimal temperature (To, blue arrow), indicating maximum oxygen delivery for growth, reproduction, and immune capacity. AS decreases in the Pejus range (Tp, orange arrows) and reaches minimal levels at the critical temperatures (Tc, red arrows).

The OCLTT hypothesis states that the ability of ectotherms to tolerate heat is limited by their ability to supply oxygen to their tissues at various temperatures set by the capacity of the cardiovascular and respiratory systems. This process produces ATP through the electron transport chain, which enables energy distribution to the cells and helps maintain metabolic fitness and immunological processes (Pörtner, Bock, and Mark 2017). Aerobic efficiency is maximized in the optimal temperatures (To), ensuring that its oxygen supply mechanisms and metabolic demands are in equilibrium and supporting the best performance of physiological functions, including immune response. Thermal limitations may commence at the transition from the optimum to the Pejus temperature (Tp)—a phase marked by progressively deteriorating conditions. This transition is characterized by a decline in oxygenation attributed to reduced ventilatory and cardiac performance. Warming beyond the point of Tp leads to a transition from aerobic to anaerobic metabolism, i.e., the critical temperature (Tc) (Dong et al. 2011; Frederich and Pörtner 2000; Pörtner and Peck 2010; Pörtner 2010) (Figure 1).

The difference between the energetic cost of the basal‐standard metabolic rate (SMR) and the maximum metabolic rate (MMR) is the highest metabolic rate an organism can support (Pörtner 2002, 2012; Pörtner and Peck 2010). This difference is known as the aerobic scope (AS) (Fry 1971, 1947), which is the portion of energy left after the basal maintenance cost. Under the OCLTT hypothesis, an organism in the optimal temperatures (To) maximizes its AS, ensuring efficient oxygen supply. The immune system is a crucial element for species maintenance and survival against infections, wound healing, cellular regeneration (Imperadore et al. 2019), and transfer of protection to progeny (Lanz‐Mendoza and Contreras‐Garduño 2022), contributing significantly to populations' viability as it recognizes and neutralizes self‐ and non‐self‐ molecules (Lanz‐Mendoza and Contreras‐Garduño 2022; Pascual et al. 2019).

Cephalopods are ectothermic mollusks characterized by their reliance solely on an innate immune response, lacking the adaptive immune system found in vertebrates. Their immune response is characterized by cellular response and humoral response, elements that form their innate immune system. The cellular response involves circulating hemocytes in the hemolymph through their closed circulatory system. The humoral response is shaped by soluble molecules such as antimicrobial peptides, components of the phenoloxidase system (PO), and coagulation pathway, lectins, reactive oxygen species, reactive nitrogen species, chemokines, and cytokines (Castellanos‐Martínez and Gestal 2013). The activity of lectins is assessed through the hemagglutination titer test, which measures their ability to agglutinate red blood cells (Abraham et al. 2022). This process is crucial for neutralization and opsonization, enhancing the functions of hemocytes. Additionally, lysozymes are proteins that exhibit lytic and isopeptidase activity against a broad spectrum of pathogens, aiding in the breakdown of bacterial cell walls and pathogen clearance (Li et al. 2019).

The relatively new eco‐immunology field is a discipline that has an integral approach through studies of immune function in relation to evolution, ecology, and physiology and predicts how environmental changes affect immunity and host–parasite interactions at a population level (Ferguson, Kortet, and Sinclair 2018; Matozzo 2016). Cephalopods are sensitive to environmental stressful stimuli (Castillo, Salazar, and Joffe 2015). These physiological stresses have several grades of response, including the secretion of hormones, changes in metabolism, immune response and behavior (Vizcaíno et al. 2023), and increased or decreased phagocytosis rate (Malham, Runham, and Secombes 1997). However, studies relating the eco‐immunology, particularly on climate change scenarios, are lacking. Nevertheless, one recent study showed that increased pCO2 led to elevated numbers of circulating hemocytes in *Octopus rubescens* , indicating a stress response and increased phagocytosis; however, no discernible effect of temperature was found (Culler‐Juarez and Onthank 2021).

The immune system is considered energetically costly (Ardia et al. 2012; Catalán et al. 2012; Sadd and Schmid‐Hempel 2009; Schmid et al. 2008) due to processes such as ATP‐dependent signaling pathways, gene expression, cell mobilization, synthesis of new molecules like antimicrobial peptides, and hematopoiesis, among other physiological processes (Ferguson, Kortet, and Sinclair 2018; Haine et al. 2008). Considering this, there is a potential trade‐off where increased metabolic demands for thermal tolerance may limit the energy available for immune system functions in cephalopods. This scenario suggests that under warmer conditions, less energy could be allocated to support the immune response mechanisms, such as cellular defense and humoral responses, potentially compromising their ability to combat pathogens effectively (i.e., immunocompetence).

We propose the hypothesis that temperature could play an essential role in controlling the immunocompetence of wild cephalopods, and this could be explained by following the ideas of the OCLTT hypothesis via AS. The energy allocation to immunocompetence activity may be compromised as the SMR exceeds optimal temperatures (i.e., stressful temperatures or Tp). We considered data from laboratory measurements and explored its relationship with the immune system metrics of wild octopuses. The mayan octopus was chosen for this study due to its accessible literature and infrastructure that allow for comprehensive information on this species (Ángeles‐González et al. 2021; Meza‐Buendía et al. 2021; Pascual et al. 2019; Rosas et al. 2014).

When it comes to cephalopods, their immune response involves both cellular (hemocytes) and humoral mechanisms (lysozyme activity PO and hemagglutination titer) that require a large energy budget to mobilize resources and synthesize molecules. This may indicate the organism's immunity strength (Mydlarz, Jones, and Harvell 2006). To understand an organism's response to environmental challenges, we must consider common types of immune responses, including those involving immune cells and humoral immune response effectors. Data on all the immune mechanisms above were obtained from the works of Pascual et al. (2019, 2020) in addition to gonadosomatic index (GSI) data. We hypothesize that thermally stressful environments will negatively associate with immunological parameters as it indicates that the capacity of energy allocation to fitness‐related processes is compromised. This information could be essential considering that cephalopods are affected by various pathogens, mainly bacteria, protozoa, and metazoan parasites (Castellanos‐Martínez and Gestal 2013).

## 2 Materials and Methods

### 2.1 Study Area

The Yucatan Peninsula (YP) is in the southeastern Gulf of Mexico. This region presents an extensive shallow continental shelf (Monreal‐Gómez, Salas‐de León, and Velasco‐Mendoza 2004) known as the Campeche Bank, in which oceanographic conditions vary widely spatially and temporally, with temperatures in the western region oscillating between 22°C and 30°C. In contrast, in the northeastern part of the YP, the Yucatan Current (YC) generates upwellings when traversing through the Yucatan Channel due to its shallow topography supplying cold water to the shelf, mainly in its eastern region. The upwelling is more noticeable during the spring and summer in the northern areas of the YP, keeping the surface temperatures at about 22°C–28°C (Enriquez and Mariño‐Tapia 2014; Merino 1997; Monreal‐Gómez, Salas‐de León, and Velasco‐Mendoza 2004).

**FIGURE 2:**
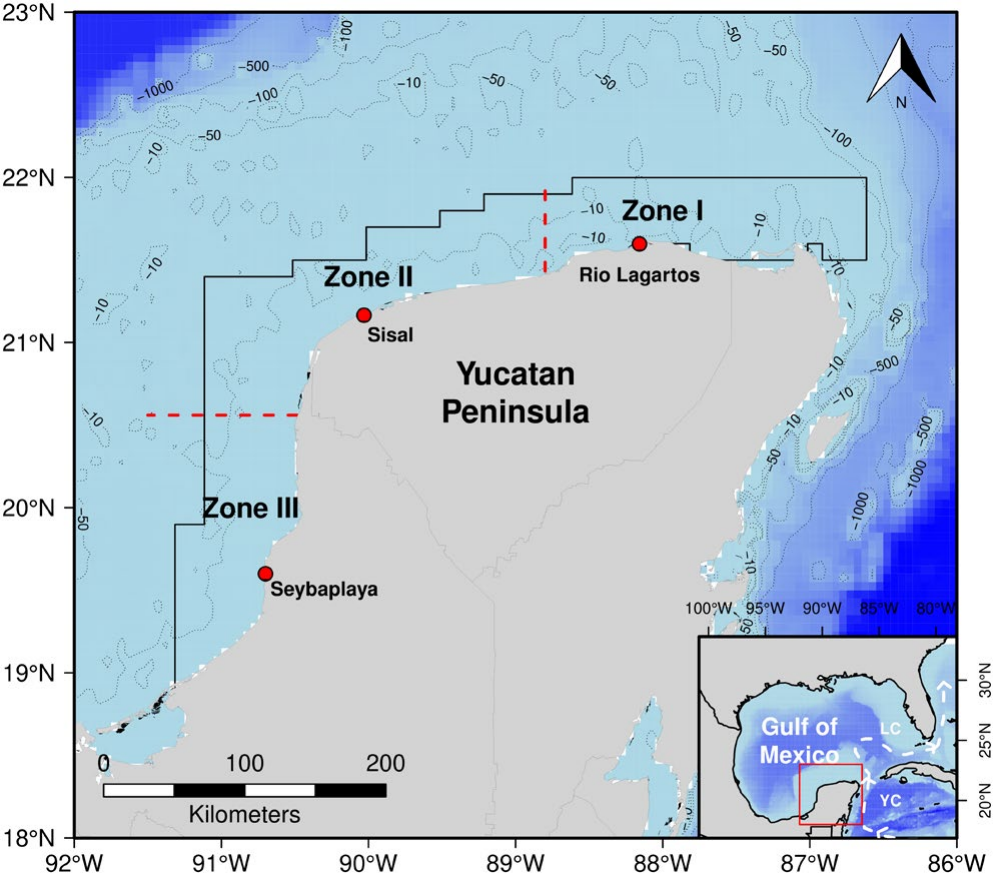
Yucatan Peninsula (YP). The polygon shows small‐scale fleet's usual fishing area of the endemic mayan octopus ( *Octopus maya* ) according to Gamboa‐Álvarez et al. (2015). Red dots indicates landing ports where organisms were collected. The Yucatan Current (YC) and Loop Current (LC—inset map) generate a northern upwelling. The polygon represents the majority of the known fishing region of the mayan octopus, although it has also been reported in deeper waters (Avendaño et al. 2019).

A study of the mayan octopus by Angeles‐Gonzalez et al. (2017) showed that YP populations could be divided into three zones according to the upwelling influence. In their work, the northeastern region was considered Zone I (Z‐I), where the upwelling influence on temperatures is easily noticed. Zone II (Z‐II) was a transitional area encompassing ports like Progreso and Celestun, experiencing a decrease in the effects of upwelling. In Zone III (Z‐III), the westernmost region, the northeast upwelling does not have an impact. The temperature changes seasonally, starting at around 24°C and reaching maximum values of approximately 30°C during the summer (Figure 2).

### 2.2 Physiological Data of Mayan Octopus

Overall, experiments have shown that this tropical endemic species, mayan octopus, is relatively sensitive to high temperatures, which is somewhat counter‐intuitive for a tropical species. According to Meza‐Buendía et al. (2021), octopuses acclimated to 30°C showed a lower thermal metabolic scope (TMS—a proxy of the AS), with higher levels of antioxidants observed in the branchial hearts. These findings suggest that temperatures above 30°C may limit energy production due to the reduced capacity of octopuses to transport oxygen to mitochondria. They hypothesize that animals are adapted to satisfy their basic energy requirements at 30°C; however, it is not enough to cover all the energy demands needed for reproduction.

Optimal TMS values were recorded at 24°C and 26°C, but beyond 26°C, TMS decreases rapidly at 27°C. This may also partly explain the reduction in reproductive performance in terms of the production of eggs, production of viable sperm, and, therefore, the quality of the progeny reported at those temperatures. Indeed, not only adults are affected but also embryos and juveniles are relatively sensitive to higher temperatures (Ángeles‐González et al. 2021; Caamal‐Monsreal et al. 2016; Juárez et al. 2015; Meza‐Buendía et al. 2021; Sanchez‐García et al. 2017). For example, at temperatures above 27°C, the number of spawned and fertile eggs is lower than at 24°C (Juárez et al. 2015), while embryonic development is also compromised above 27°C.

Based on this information and the TMS data from Meza‐Buendía et al. (2021), we consider temperatures above 27°C stressful (referred here as “thermally stressed”). Conversely, mayan octopus appears to tolerate lower temperatures well for a tropical species, with juvenile TMS indicating that temperatures as low as 22°C can be optimal (Ángeles‐González et al. 2021) and good embryo development occurring at 22°C (Caamal‐Monsreal et al. 2016). The fishing areas of the YP (Figure 2) rarely experience temperatures lower than 22°C, so we have considered any temperature below 27°C as favorable for the species (referred as “non‐stressed”).

### 2.3 Catch and Equipment for Mayan “Octopus” Immunological Measurements

#### 2.3.1 Octopus Sampling

The organisms came from two collection periods: the first was designed to determine seasonal variation, and the second to assess the effect of seasonal upwelling on the immune status of adult octopuses (Pascual et al. 2019, 2020). Briefly, between May 2007 and February 2008, wild adult organisms were captured in the coastal areas of Sisal, Yucatan, Mexico (21°09′ N; 90°01′ W; Z‐II), in three different climatic seasons and were assigned based on local seasonality: (1) the winter season (February), (2) the dry season (May), and (3) the rainy season (September). The second group was collected at three locations from February to July, 2010: Ría Lagartos (21°38′ N, 88°10′ W) located in the upwelling zone (Z‐I); Seybaplaya (19°38′ N, 90°41′ W) corresponding to a zone with no influence of upwelling (Z‐III); and Sisal (21°09′ N, 90°01′ W), a zone of transition located between the two former (Z‐III).

Organisms were caught using the “gareteo” technique (a local drift‐fishing method) that allows catching the organisms without harming them. On board, organisms were placed inside a black tank with 1000 L of sea water in constant replacement by using a submersible pump. Transportation from the capture areas to the laboratory facilities took 2–4 h. Organisms were settled in individual green tanks (45 cm in diameter, 60 cm in depth, with 80 L of seawater) in a closed area with controlled photoperiod (12:12 light–dark), along with a constant aeration system and daily seawater flow exchange equivalent to 300% (26°C ± 1°C, 37 ± 1 PSU, pH 8.1 ± 0.2). Samples were obtained the next day, and organisms were not fed to avoid influencing the immunologic indicators and physiological status.

Before hemolymph sampling, octopuses were reassured by hypothermia. To achieve this, organisms were individually immersed in cold water (17°C below the maintenance system temperature) for 2–4 min (Cruz‐López 2010; Roumbedakis et al. 2018). When respiratory rate and locomotor activity decreased, and the response to a contact stimulus was absent, the animals were removed, and hemolymph was withdrawn from the aorta with a cold sterile catheter connected to an Eppendorf tube (Cruz‐López 2010). To avoid immune system activation by endotoxins, all glassware was washed with Etoxa‐clean before use, and solutions were prepared using pyrogen‐free water and filtered through a 0.2‐μm Acrodisc.

#### 2.3.2 Immunological Variables

Hemolymph was centrifuged at 800 *g* for 5 min at 4°C to separate the plasma, which was used to evaluate PO, lysozyme activity, and hemagglutination activity. The cellular pellet was washed twice with isotonic solution (IS: 0.45 M NaCl, 10 mM KCl, 10 mM HEPES, 7.3 pH, and 10 mM EDTA–Na2) and centrifuged again. Then, the cellular pellet was re‐suspended several times with cacodylate buffer (10 mM cacodylic acid, 10 mM CaCl, pH 7.0) in equal volume of hemolymph and centrifuged at 13,000 *g* for 5 min at 4 °C. The supernatant was used to evaluate the PO activity from degranulated hemocytes. PO was measured in 96‐well flat bottom plates (Hernández‐López et al. 1996).

The technique was adjusted for mayan octopus (Roumbedakis et al. 2018). First, 50 μL of the plasma and degranulated hemocytes were incubated for 10 min at 37°C to transform proPO into PO without using exogenous trypsin. Then, 180 μL of L‐3,4‐dihydroxyphenylalanine (L‐DOPA, 3 mg/mL; Sigma D9628) was added to each well and the microplate was incubated for more than 10 min at 37°C. Absorbance was measured at 490 nm in a microplate reader (Benchmark Plus BioRad). Evaluations were performed in triplicate, and results were expressed as an increment of 0.001 in optical density.

The THC were counted in a Neubauer chamber from a hemolymph aliquot fixed with 10% formaldehyde in Alsever solution (115 mM C6H12O6, 30 mM Na3C6H5O7, 338 mM NaCl, 10 mM EDTA–Na2, pH 7.0) with a 1:3 dilution (Roumbedakis et al. 2018). Counting was performed in duplicate, covering a minimum area count of 0.04 cubic millimeters and expressed as cells/mm^3^. Hemagglutination activity was measured using human blood (type O+). 50 μL of octopus plasma was added to a U‐shaped 96‐well microtiter plate, and a twofold serial dilution was prepared using a 0.9% saline solution as the diluent. In controls, plasma was replaced by 0.9% saline solution. Plasma hemagglutination titer was expressed as the reciprocal of the highest dilution, showing a positive visible pattern of agglutination (Pascual‐Jimenez et al. 2012).

Lysozyme activity was quantified according to the turbidimetric method of Parry et al. (1965) with slight modification. *Micrococcus* was suspended in 0.05 M sodium phosphate buffer (pH 7.3), transferred to a cuvette, and read at a UV–vis spectrophotometer at 530 nm. 100 μL of plasma was transferred to the cuvette, and the reduction in absorbance was recorded. The result was expressed as U/mL (Pascual et al. 2019) (Appendix S1).

### 2.4 Environmental Layer Data and Field Aerobic Scope Database

This study has been conducted using E.U. Copernicus Marine Service Information (https://www.copernicus.eu/en); specifically, we used the daily bottom temperatures from the GLORYS12V1 product (https://doi.org/10.48670/moi‐00021) for the periods 2007–2008 and 2009–2010. With the GLORYS12V1 data, we projected the daily bottom temperature maps (resolution of ~9 × 9 km). We also masked the bottom temperature map up to a 30‐km distance up the shore using (resolution of ~1 × 1 km) the marspec environmental layer (Sbrocco and Barber 2013). The distance up the shore was resampled to the same resolution of daily bottom temperatures.

A database was created that incorporated both immunological variables and the median temperature, taking into account the day of observation and the sampling areas (Z‐I, Z‐II, and Z‐III). For instance, if an octopus was caught in June in the Z‐I, we used the corresponding polygon from Figure 2 to calculate the median bottom temperature. The temporal match was done following the methodology of Torrejón‐Magallanes et al. (2021). All this process was done using the software R (R Core Team 2023).

### 2.5 Statistical Analyses

The organisms (*n* = 286) were categorized into stressed (> 27°C) and non‐stressed (< 27°C) according to the median bottom temperature corresponding to the date and fishing region calculated previously. We explored the immunological data (THC [*n* = 282], hemagglutination [*n* = 117], lysozyme activity [*n* = 117], and PO patterns [*n* = 279]) with boxplots to graph the immunological data regarding the octopus's thermal stress condition to detect potential patterns. Additionally, we present the median and interquartile range (IQR) values. We also evaluated the immunological data between thermally stressed and non‐stressed animals using a Mann–Whitney *U* test. Later, we conducted a nonparametric multidimensional scaling (nMDS) with the immunological variables scaled and centered with the “vegan” library (Oksanen et al. 2022) in R. To further explore the relationship between thermally stressed and non‐stressed animals and their immunological parameters, we utilized the “envfit” function from the vegan package. This function fits vectors to the nMDS, projecting points onto the vectors that exhibit the highest correlation with the chosen variables (i.e., THC, lysozyme activity, PO, and hemagglutination).

To address the significant spatiotemporal variations in maturation, growth, reproduction, juvenile, and reproductive aggregations observed in the YP (Angeles‐Gonzalez et al. 2017; Markaida, Méndez‐Loeza, and Rosales‐Raya 2017), we employed generalized linear mixed models (GLMM) via the “glmmTMB” library (Brooks et al. 2017) with all the immunological variables. We considered the maturity of organisms (mature and immature) and sex (male and female) based on measurements of the GSI. Criteria for maturity were established following a review of the work by Avila‐Poveda et al. (2016). For females, maturity was defined by the presence of mature gametes, specifically vitellogenic oocytes, with a GSI cutoff of 2. Males were considered mature if they had a GSI of 0.7 or higher, indicating the formation of spermatozoa ready for reproduction. This is important since size and age can affect cephalopod immunological parameters (White et al. 2023). Using the performance library (Lüdecke et al. 2021) in R, we computed the marginal *R* ^2^ (variance explained by fixed effects) and conditional *R* ^2^ (variance explained by the entire model, including fixed and random effects).

The assumptions of the GLMM were evaluated using the DHARMa package (Hartig 2022) conducting the following assessments: a Kolmogorov–Smirnov test (KS test) for uniformity of the simulated residuals, tests for dispersion, an outlier test, and residuals versus predicted values to assess the reliability and validity of the results. The assessment of the KS test (*p* = 0.63), the dispersion test (*p* = 0.99), and the outlier test (*p* = 1), combined with a visual inspection of the residuals versus predicted values, indicates that our GLMM model was reliable and meets the necessary assumptions for valid inference (Figure S1).

## 3 Results

**FIGURE 3:**
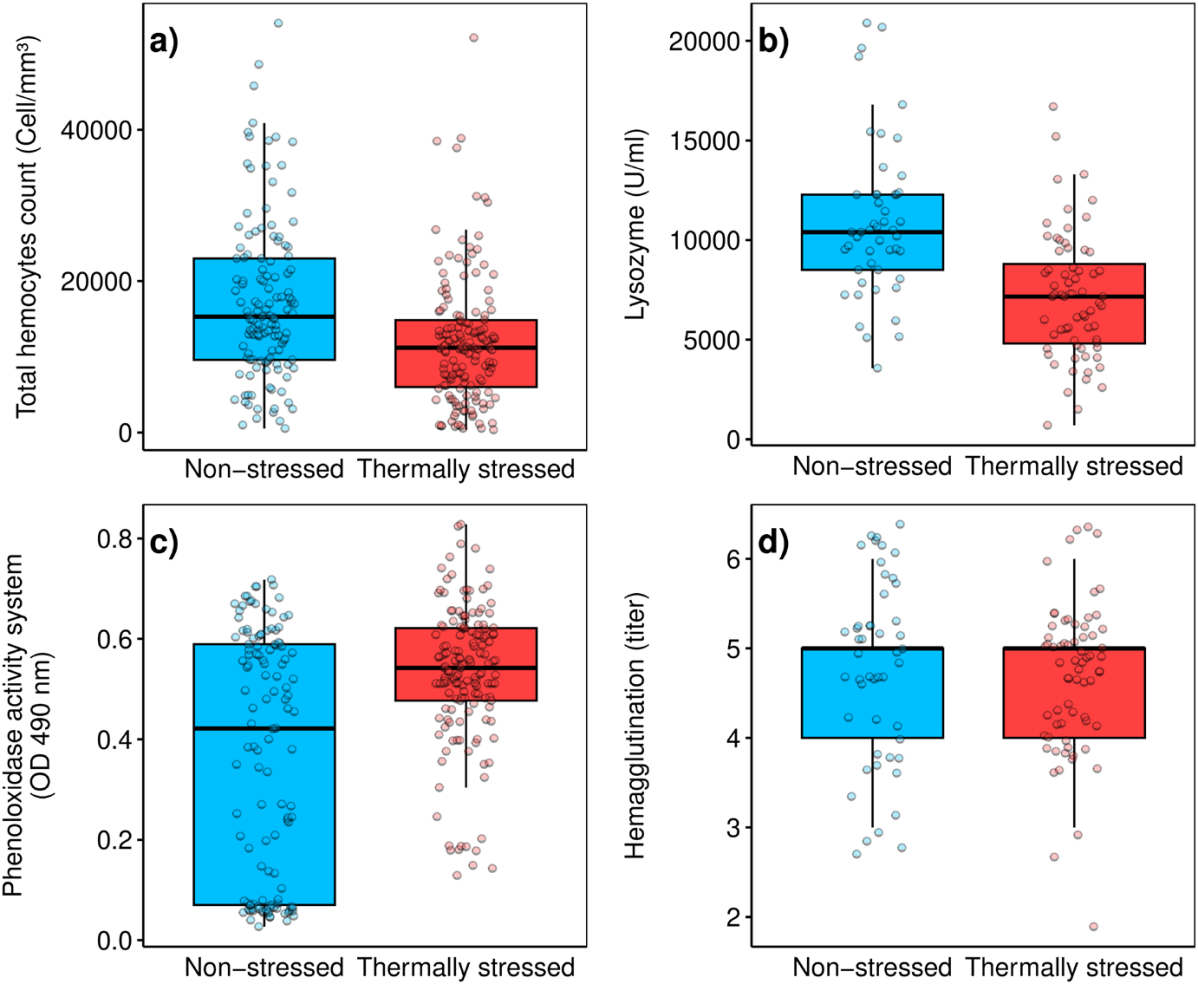
The immunological variables of the mayan octopus ( *Octopus maya* ) divided by non‐stressed and stressed organisms according to the average temperature, where temperatures above 27°C are considered as thermally stressed organisms.

Boxplots indicate that lower thermal stress levels are associated with higher THC levels (Figure 3a). Non‐stressed organisms exhibit a median THC of 15,300 (IQR: 9600–23,000) cells/mm^3^, while stressed organisms show a median of 11,200 (IQR: 6000–14,850) cells/mm^3^. Similarly, lysozyme activity is higher under non‐stressful temperatures, with a median of 10,395 (IQR: 8505–12,285) U/mL, compared to a median of 7163 (IQR: 4812–8800) U/mL in stressful conditions (Figure 3b). In contrast, thermally stressed octopuses display higher PO levels, with a median of 0.5420 (IQR: 0.47–0.62) OD 490 nm, compared to non‐stressed octopuses, which have a median of 0.42 (IQR: 0.07–0.58) OD 490 nm (Figure 3c). No clear pattern was observed for hemagglutination, as the values were similar for both stressed and non‐stressed organisms (median: 5, IQR: 4–6 titer) (Figure 3d). According to the Mann–Whitney *U* test, statistical differences are found for THC, lysozyme activity, and PO (*p* < 0.001) but not for hemagglutination (*p* = 0.332).

**FIGURE 4:**
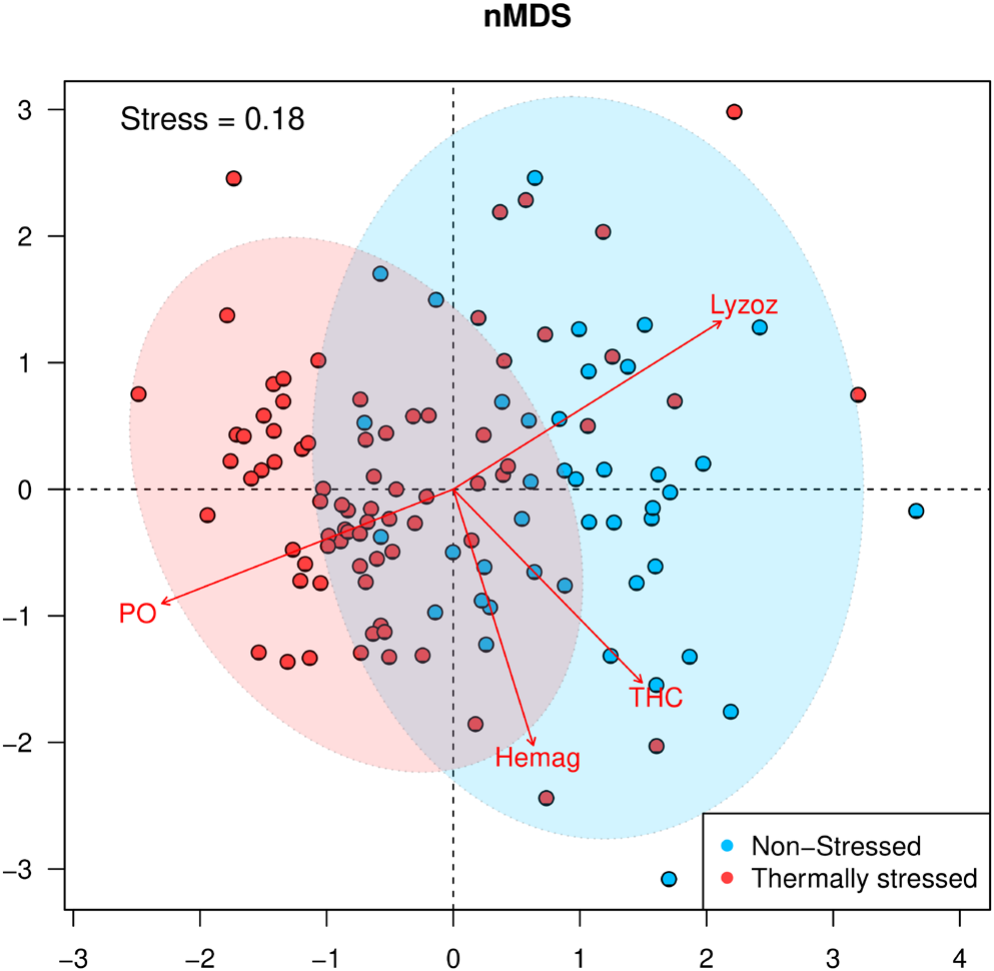
Immunological variables of the mayan octopus ( *Octopus maya* ) were analyzed using nonparametric multidimensional scaling. The data were categorized in non‐stressed (< 27°C) and thermally stressed organisms (> 27°C). The ellipses shown encompass 95% of the data.

The nMDS analysis had a stress of 0.18, suggesting a good representation of the multivariate data structure. The nMDS showed that the organisms were arranged according to the stressed and non‐stressed conditions, although an overlap between values was observed (Figure 4). According to the envfit function, there was a statistically significant correlation (*p* < 0.001) between all immunological variables and the ordination. Specifically, the lysozyme activity strongly correlates with non‐stressed animals, while THC and hemagglutination exhibited a weaker association. In contrast, PO was primarily linked to stressed organisms.

The results of the GLMM indicated a marginal *R* ^2^ of 0.79 and a conditional *R* ^2^ of 0.84. Statistically significant effects were found for THC (estimate = −0.0001, standard error = 0.00004, *p* = 0.032) and PO (estimate = 17.1, standard error = 3.64, *p* < 0.001). In contrast, the lysozyme activity (estimate = −0.0001, standard error = 0.0001, *p* = 0.137) and hemagglutination (estimate = −0.45, standard error = 0.51, *p* = 0.372) were not significant.

**FIGURE 5:**
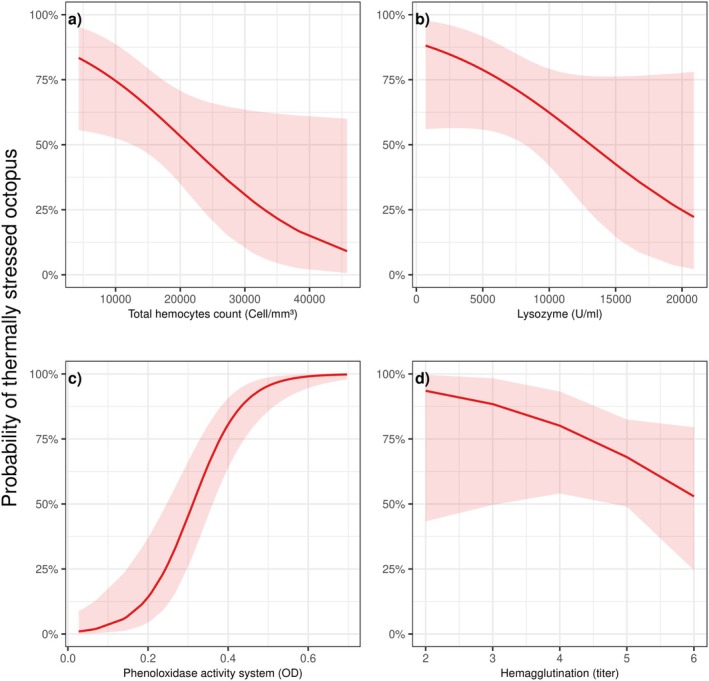
Relationship between thermally stressed mayan octopuses ( *Octopus maya* ) and immunological parameters: Total hemocyte count (a), lysozyme activity (b), phenoloxidase activity (c), and hemagglutination (d). Statistical significance was observed for total hemocyte count and phenoloxidase activity.

Low THC levels of 4300 cells/mm^3^ indicated a high probability of thermal stress at 83.4% (standard error = 0.00004; 95% CI: 0.55–0.95), whereas higher THC levels of 45,800 cells/mm^3^ resulted in a significantly lower probability of thermal stress at 9% (standard error = 1.38; 95% CI: 0.006–0.59) (Figure 5a). Although lysozyme activity did not show statistical significance (Figure 5b), lower levels (700 U/mL) were associated with a higher probability of thermal stress at 88% (standard error = 0.90; 95% CI: 0.55–0.97), decreasing to 22% (standard error = 1.28; 95% CI: 0.02–0.77) at higher lysozyme activity (20,895 U/mL).

For PO, the lowest values (0.02 OD 490 nm) corresponded to a nearly 0% probability of thermal stress at 0.01% (standard error = 1.14; 95% CI: 0.001–0.08). Conversely, the probability of thermal stress increased to 99% (standard error = 1.20; 95% CI: 0.97–0.99) at higher PO values of 0.69 OD 490 nm (Figure 5c). While hemagglutination results (Figure 5d) were not statistically significant, trends suggested that lower hemagglutination levels (titer of 2) were linked to a probability of thermal stress of 0.93% (standard error = 1.49; 95% CI: 0.43–0.99), compared to a lower probability of 52% (standard error = 0.63; 95% CI: 0.24–0.79) at hemagglutination levels with a titer of 6.

## 4 Discussion

### 4.1 Temperature Stress and the Immune System of the Mayan Octopus

The impact of environmental conditions can alter the immune system, providing insights into physiological health (Castillo, Salazar, and Joffe 2015). For example, Pascual et al. (2019) found that mayan octopuses in warmer waters exhibited metabolic stress and immunological compensation mechanisms through hematological markers. This is demonstrated by heightened PO, which shows higher values of activity detection. Additionally, the low levels of lysozyme activity suggested that the increased PO was not due to bacterial presence in the hemolymph but possibly linked to cellular stress such as oxidative stress.

Interestingly, there appears to also be a positive correlation between non‐stressing temperatures and THC. These findings differ from those of López‐Galindo et al. (2019), who found that mayan octopuses exposed to 30°C showed increased THC levels. One potential explanation for this difference is that higher temperatures can speed up biochemical processes such as protein synthesis and cellular migration (Haynie 2001), at least in the short term. However, maintaining strong immunity over long periods requires a lot of energy and can be challenging (Haine et al. 2008; Moret and Schmid‐Hempel 2000). Hence, optimal immunity may not only depend on a specific environmental temperature but also on having enough energy, which is influenced by the prevailing environmental conditions (Catalán et al. 2012; Li et al. 2007). Such patterns in immune system parameters in mayan octopuses can be elucidated through the OCLTT hypothesis and the AS.

The thermal tolerance is constrained by the efficiency of its cardiovascular and respiratory systems in delivering oxygen and producing ATP under varying temperature conditions (Pörtner and Peck 2010). Considering that the immune system is energetically costly (Ardia et al. 2012; Catalán et al. 2012; Sadd and Schmid‐Hempel 2009; Schmid et al. 2008), our results could show that when energetic demands increase to satisfy basic energy requirements due to increasing temperatures—such as basal metabolism, antioxidant defense mechanisms, activity, and growth (Frederich and Pörtner 2000; Meza‐Buendia et al. 2024; Meza‐Buendía et al. 2021)—there may not be sufficient energy allocated to the immune system. This would compromise the immunocompetence of the mayan octopus since it creates trade‐offs between components of the immune system (Ardia, Parmentier, and Vogel 2011; Rao, Ling, and Yu 2010) or life history traits (Li et al. 2007; Moret and Schmid‐Hempel 2000; Rantala and Roff 2005; Semmens et al. 2004).

Illustrative examples of these concepts include the observed shift in growth curves from exponential to power functions in cephalopods due to increased energy demands in the gonads, representing an adaptation in which energy is redirected from somatic growth to reproductive processes (Ho, Moltschaniwskyj, and Carter 2004; Moltschaniwskyj 2004; Semmens et al. 2004). Another study on female mayan octopuses' health status on different days post‐spawning indicated that reserve consumption coincided with heightened immune responses, characterized by increased hemagglutination and PO. This suggests that females may be compensating immunologically to care for their eggs until hatching (Roumbedakis et al. 2018).

Octopuses under thermal stress (at Tp or nearby Tc—Figure 1) might be prioritizing the activation of the PO, even if it involves energetically costly processes like melanin synthesis and oxidative stress responses (Castillo, Salazar, and Joffe 2015; Dubovskii et al. 2010) over the THC. While temperature experiments have shown increased THC for cephalopods (Culler‐Juarez and Onthank 2021), including the mayan octopus (López‐Galindo et al. 2019), these experiments typically involve relatively short‐term measurements. In contrast, wild octopuses must cope with consistent stressful temperature conditions and face energetic trade‐offs between health parameters and life history traits, which could create discrepancies between experimental data and wild observations. Similar patterns have been observed in other species (Rolff 2001; Schmid et al. 2008).

In contrast, lower PO levels have been observed in organisms with more favorable physiological and nutritional indicators, such as greater reserves and higher metabolite concentrations in the hemolymph and digestive gland. This suggests that these organisms may be less susceptible to opportunistic infections, leading to reduced activation of the immune system (Pascual et al. 2019, 2020).

Lysozyme activity levels were not statistically significant in the GLMM. However, exploratory plots and the Mann–Whitney *U* test suggest that optimal oxygen delivery may enable better energetic allocation, leading to higher baseline levels of lysozyme (and thermal tolerance capacity), although the evidence is inconclusive. Lysozyme activity sometimes exhibits a contrary reaction to PO, as studies have reported negative correlations between baseline levels of antibacterial activity and cellular immunity (Ardia et al. 2012; Cotter et al. 2008).

These findings show that the thermal stress could lead octopuses to prioritize multimeric immune systems over antibacterial activity (lysozyme). The amplification of an invertebrate's immune response is associated with the PO. This innate immune reaction provides toxic quinone substances and melanin that physically encapsulate pathogens and participate in the wound healing process (Cerenius and Söderhäll 2021). Such patterns could also suggest that optimal oxygen delivery could enhance the organism's ability to defend against infections, which could indicate better immunocompetence due to the various functions that cells perform (i.e., THC), including pathogen‐associated molecular patterns (PAMPs) and damage‐associated molecular pattern (DAMP) recognition, which in turn induce a receptor‐dependent response as a wound healing, encapsulation, melanization, phagocytosis among others (Cerenius and Söderhäll 2021). Finally, the lack of a relationship between hemagglutination and AS indicates that metabolic efficiency, oxygen utilization, and other mechanisms might be in play and are not accounted for in this work.

The interactions among the central nervous system, the endocrine, and the immune systems that result in the regulation of the immune system have been well‐established in mammals (Yang and Glaser 2002). Lymphocytes, monocytes/macrophages, and granulocytes have been shown to exhibit receptors for many neurotransmitters (Felden 1991). It has been described that catecholamines can indirectly cause changes in immune activities such as lymphocyte trafficking and proliferation, antibody production, and cell lysis through the regulation of cAMP levels (Madden and Livnat 1991). Alterations in the immune response due to stress have barely been studied in marine intervertebral groups from an ecological perspective (Ellis et al. 2011). The results of this research provide information to increase our understanding of the ecological significance of environmental stressors on the immune status of octopuses.

### 4.2 Biogeographical and Climate Change Implications

Usually, macroecology processes are described by correlative analyses under a top–bottom approximation; however, as helpful as they may be to test hypotheses (e.g., Angeles‐Gonzalez et al. 2024; Ochoa‐Zavala et al. 2022; Osorio‐Olvera et al. 2020), they do not allow for an understanding of the process behind them (Connolly et al. 2017; Keith et al. 2012). Such limitations have made some researchers favor mechanistic approaches (Connolly et al. 2017; Kearney and Porter 2004; Keith et al. 2012). Works describing the usefulness of the AS detailing wild population characteristics exist (Angeles‐Gonzalez et al. 2023; Payne et al. 2016; Pörtner and Knust 2007). For example, Pörtner and Knust (2007) showed that the oxygen limitation in *Zoarces viviparus* matched temperatures where growth performance and abundance decreased.

Given past studies and the results of this study, OCLTT and, by extension, AS could serve as valuable tools for monitoring populations. Specifically, they could be used to assess key population traits related to fitness, such as immune status, to detect regions susceptible to low immunocompetence and thus disease outbreaks and potentially infer changes under climate change. Indeed, increased temperatures, coupled with stressors such as deoxygenation or acidification affecting the AS (Pörtner and Peck 2010), could detrimentally impact the physiological and immunological systems of mayan octopus populations.

Parisi et al. (2017) suggested that this could lead to disease outbreaks, creating a cycle where organisms struggle to mount an effective immune response, leading to deterioration in body condition. In this sense, physiological data may be useful to detect spatially vulnerable populations to disease outbreaks. Nevertheless, it is important to acknowledge that our models may not explain all the observed variance, since many other factors also likely influence organism distribution and health, including demography, seasonality, biotic interactions, foraging behavior, dispersal capacity, Allee effects (Dallas and Santini 2020; Osorio‐Olvera et al. 2020; Osorio‐Olvera et al. 2019), ontogenetic development, and environmental conditions beyond temperature alone (Pörtner and Peck 2010).

## 5 Conclusions

Our research suggests that physiological data from laboratory studies can play a crucial role in predicting the fitness of organisms in response to environmental factors like temperature. This work has shown a potential connection between thermal stress and immunocompetence, which could impact population processes such as increased susceptibility and incidence of infectious diseases. These findings are also significant for understanding the mayan octopus's dynamics in field data. Considering this, physiological data can provide quick evaluations of environmental conditions and their suitability for supporting population health. Further testing of our work's hypothesis is necessary to understand whether the association seen here is the rule or not.

## Author Contributions

**Luis Enrique Angeles‐Gonzalez:** conceptualization (equal), data curation (equal), formal analysis (equal), methodology (equal), writing – original draft (equal), writing – review and editing (equal). **Laura Alvarez‐Lee:** conceptualization (equal), data curation (equal), methodology (equal), writing – original draft (equal), writing – review and editing (equal). **Luis Osorio‐Olvera:** methodology (equal), software (equal), supervision (equal), writing – review and editing (equal). **Estefany López‐Ripoll:** data curation (equal), writing – original draft (equal), writing – review and editing (equal). **Fernando Díaz:** conceptualization (equal), writing – original draft (equal), writing – review and editing (equal). **Carlos Rosas:** conceptualization (equal), methodology (equal), writing – original draft (equal), writing – review and editing (equal). **Honorio Cruz‐López:** data curation (equal), methodology (equal), writing – review and editing (equal). **Cristina Pascual:** conceptualization (equal), data curation (equal), formal analysis (equal), resources (equal), supervision (equal), writing – original draft (equal), writing – review and editing (equal).

## Conflicts of Interest

The authors declare no conflicts of interest.

## Supporting information


Appendix S1.

Figure S1.

## Data Availability

The data and scripts for this study replication are available in Figshare (https://doi.org/10.6084/m9.figshare.24257545.v1).
