# Supplementary material for: Linking Inferred Laboratory‐Derived Temperature Stress to the Immunocompetence of Wild Octopus maya (Mayan Octopus) G.L. Voss & Solís, 1966
Source: Ecol Evol. 2025 Mar 19;15(3):e70805. doi: 10.1002/ece3.70805 (PMC11922577; doi:10.1002/ece3.70805)
Supplement: Supplementary file 2 — Figure S1. [file ECE3-15-e70805-s001.docx]

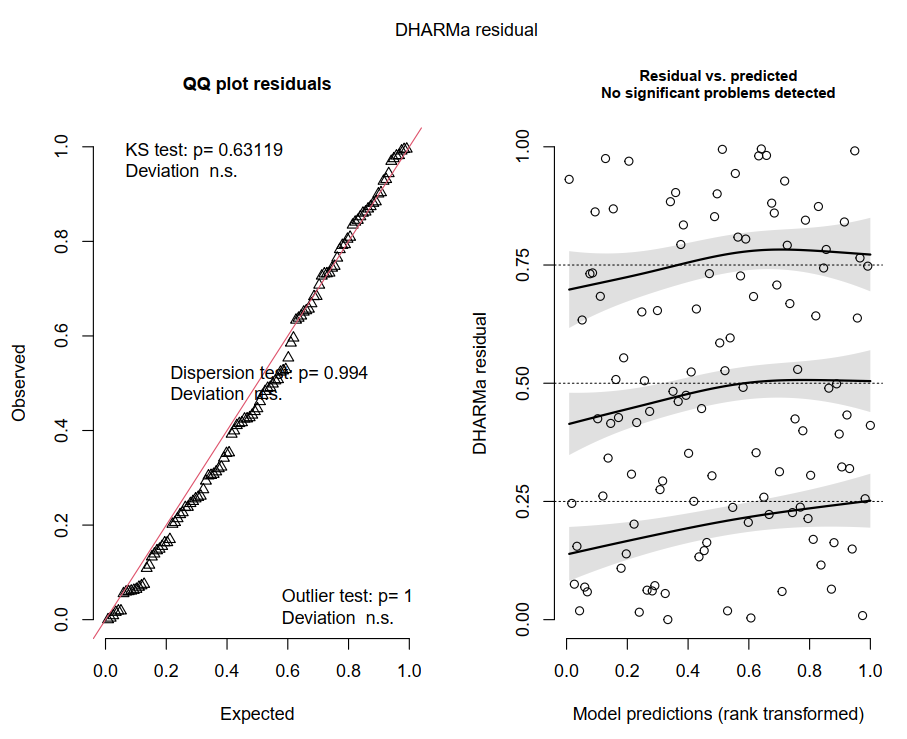


**Figure S1.** Diagnostic plots of simulated residuals from the GLMM model. The plots assess the model's assumptions, including homoscedasticity, normality, and independence. All assumptions were satisfied, indicating an appropriate model fit.
